# An optogenetic analogue of second-order reinforcement in *Drosophila*

**DOI:** 10.1098/rsbl.2019.0084

**Published:** 2019-07-03

**Authors:** Christian König, Afshin Khalili, Thomas Niewalda, Shiqiang Gao, Bertram Gerber

**Affiliations:** 1 Department Genetics of Learning and Memory, Leibniz Institute for Neurobiology (LIN), Brenneckestrasse 6, 39118 Magdeburg, Germany; 2 Julius-von-Sachs-Institute, University of Würzburg, Julius-von-Sachs Platz 2, 97082 Würzburg, Germany; 3 Institute for Biology, Otto von Guericke University Magdeburg, Universitätsplatz 2, 39106 Magdeburg, Germany; 4 Center for Behavioral Brain Sciences (CBBS), Universitätsplatz 2, 39106 Magdeburg, Germany

**Keywords:** *Drosophila melanogaster*, reinforcement, second-order conditioning, mushroom body, dopamine, prediction

## Abstract

In insects, odours are coded by the combinatorial activation of ascending pathways, including their third-order representation in mushroom body Kenyon cells. Kenyon cells also receive intersecting input from ascending and mostly dopaminergic reinforcement pathways. Indeed, in *Drosophila*, presenting an odour together with activation of the dopaminergic mushroom body input neuron PPL1-01 leads to a weakening of the synapse between Kenyon cells and the approach-promoting mushroom body output neuron MBON-11. As a result of such weakened approach tendencies, flies avoid the shock-predicting odour in a subsequent choice test. Thus, increased activity in PPL1-01 stands for *punishment*, whereas reduced activity in MBON-11 stands for *predicted punishment*. Given that punishment-predictors can themselves serve as punishments of second order, we tested whether presenting an odour together with the optogenetic silencing of MBON-11 would lead to learned odour avoidance, and found this to be the case. In turn, the optogenetic activation of MBON-11 together with odour presentation led to learned odour approach. Thus, manipulating activity in MBON-11 can be an analogue of predicted, second-order reinforcement.

## 1. Introduction

Animals and humans go to great lengths to obtain rewards, such as food and water, and to avoid punishment, such as bodily damage and pain. Essential to these processes is the learning of cues predictive of such actual or first-order reinforcement. Critically, predictive cues not only acquire learned valence but, once predictive relationships are established, also can confer learned valence themselves; i.e. they can serve as second-order reinforcement [1–3]. In humans, for example, learning that money can buy food establishes money as a second-order reward. In general, second-order conditioning may underlie chains of predictions and early anticipatory behaviour in humans and animals. Indeed, the capacity for second-order conditioning is widely distributed across the animal kingdom, including insects [4–7], and is implemented in many computational models of associative learning [8].

**Figure 1.**
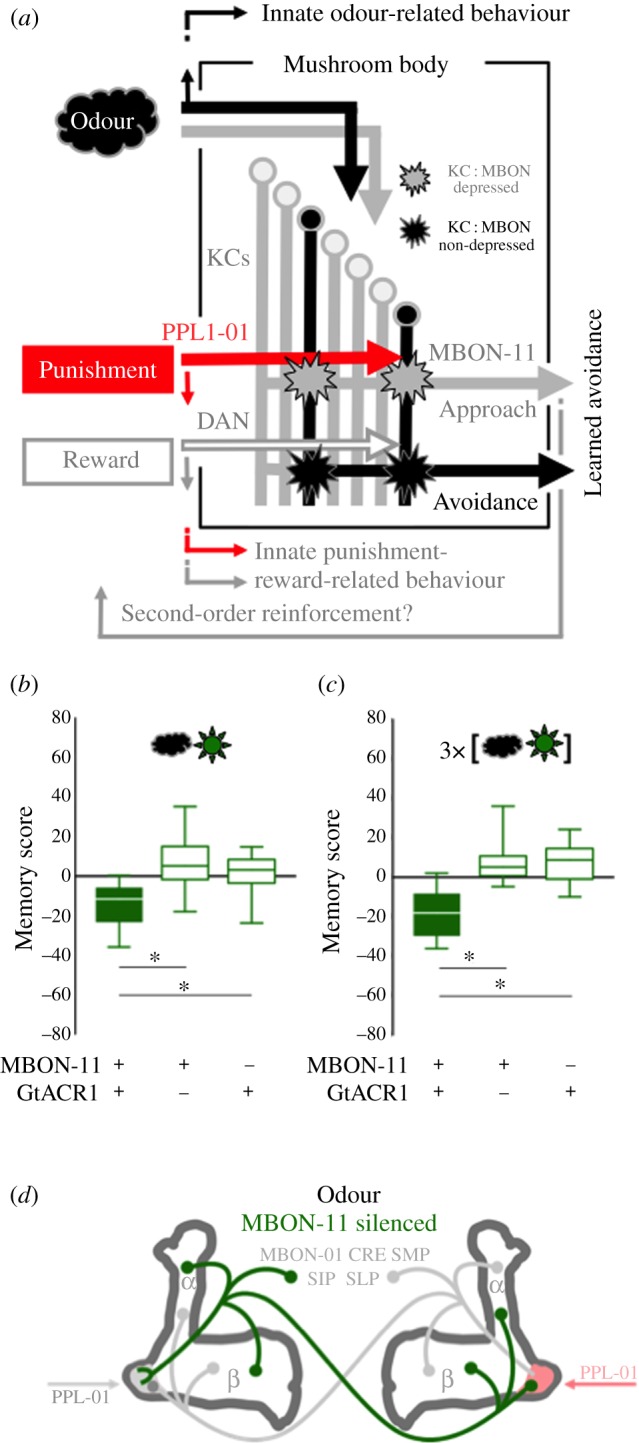
(*a*) Simplified account of odour–shock associative learning in flies (after [9–14]). Odour presentation in untrained animals mediates balanced approach and avoidance tendencies of mushroom body output neurons (MBONs). Coincidence of odour-evoked activity in the mushroom body Kenyon cells (KCs) and activity of the dopaminergic neuron PPL1-01 evoked by the electric shock leads to a depression of the synapses from these KCs to an approach-promoting MBON. In a subsequent test, this allows avoidance tendencies through non-depressed KC-MBON synapses in parallel compartments to prevail. The organization of innate olfactory, punishment- and reward-related behaviour largely bypasses the mushroom body. For simplicity KC–KC, KC–DAN, DAN–MBON and MBON–MBON synapses are omitted from this figure [15,16]. Cloud: odour; star: depressed/non-depressed KC-MBON synapse. A possible feedback from the MBONs towards the DANs is indicated. Note the multiple targets of MBON-11 within the ipsi- and contralateral mushroom body, as well as outside the mushroom body sketched in (*d*). (*b*) Presenting odour (cloud) with green light (star) leads to aversive associative memory in flies expressing the green-light-gated anion-channel GtACR1 in MBON-11, but not in genetic controls. (*c*) As in (*b*), using three training trials with an inter-trial interval of 3 min. (*d*) Sketch of connectivity of MBON-11; Greek letters refer to mushroom body lobes. Target regions of MBON-11 outside the mushroom body include MBON-01, the crepine (CRE) and the superior medial, intermediate and lateral protocerebrum (SMP, SIP, SLP) (after [13]). Postsynaptic partners of the contralateral branch of MBON-11 include PPL1-01 [17]. All these target regions could contribute to the reinforcing effects of manipulating the activity of MBON-11. Data are displayed as box plots (middle line: median; box boundaries and whiskers: 25/75% and 10/90% quantiles, respectively). Data were analysed across groups by Kruskal–Wallis tests at *p* < 0.05, followed in the case of significance by pairwise comparisons with Mann–Whitney *U*-tests at *p* < 0.05 with Bonferroni–Holm correction (asterisk). Underlying preference scores can be found in the electronic supplementary material, figure S1. Sample sizes and statistical results can be found in the electronic supplementary material, table S1. A ‘+’ below box plots indicates the presence of the respective transgene. (Online version in colour.)

In flies, presenting odour A with an electric shock punishment and odour B without punishment leads to learned avoidance of A in a subsequent choice test. This learning of an odour as a predictor of electric shock takes place in the Kenyon cells (KCs) of the mushroom body (figure 1*a*) [9–12]. The mushroom body provides a sparse, combinatorial representation of the sensory environment, including odours. Along their long axonal fibres, the KCs further receive intersecting input from neurons mediating internal reinforcement, many of which are dopaminergic (DANs). The coincidence of activation by odour and of DAN signalling can lead to presynaptic plasticity at the cholinergic synapse between the KCs and the output neurons of the mushroom body (MBONs). Arborizations from DANs and MBONs overlap and are regionally confined along the KC fibres, establishing a characteristic compartmental organization. In the case of the PPL1-01 DAN mediating an internal punishment signal, synaptic strength between the odour-coding KCs and the approach-promoting MBON-11 is reduced [18,19]. For the punished odour, the innate balance between approach and avoidance is thus tilted in favour of avoidance. In other words, activity in PPL1-01 can provide first-order punishment, and an odour that predicts first-order punishment leads to reduced activity in MBON-11. We therefore wondered whether, in experimentally naive flies, optogenetically silencing MBON-11 might be an analogue of a punishment-predicting odour such that it confers a punishing effect of second-order upon an actually present odour associated with such silencing (also see [20])—and whether in turn optogenetically activating MBON-11 might have a rewarding effect.

## 2. Material and methods

Procedures follow [21], unless mentioned otherwise. *Drosophila melanogaster* were maintained on standard food, with 60–70% relative humidity, at 25°C, and in constant darkness to prevent unintended optogenetic effects. Flies aged 1–3 days after hatching were collected and kept at 18°C for up to four additional days. MB320C and MB085C (Fly Light Split-GAL4 Driver Collection) [13] as driver strains covering the PPL1-01 and MBON-11 neurons, respectively, were crossed to UAS-ChR2-XXL (Bloomington stock number: 58374) [22] or UAS-GtACR1 as effectors for optogenetic activation or silencing, respectively. To generate the latter strain, the GtACR1 DNA was synthesized (Thermo Fisher Scientific) according to the published sequence [23] with codon usage optimized to *D. melanogaster*. The synthesized GtACR1 DNA with a C-terminal YFP was inserted into the expression vector pJFRC7. Embryo injection (BestGene Inc.) was performed to establish flies carrying UAS-GtACR1. Crosses for genetic controls yielded animals heterozygous for either construct. Synonyms for PPL1-01 are PPL1-γ1pedc and MB-MP1; synonyms for MBON-11 are MBON-γ1pedc>α/β and MB-MVP2.

Behavioural experiments used a set-up from CON-ELEKTRONIK (Greussenheim, Germany) and took place at 23–25°C and 60–80% relative humidity. Training was performed in red light, which is invisible to flies, and testing in darkness. As odorants, 50 µl benzaldehyde (BA) and 250 µl 3-octanol (OCT) (CAS 100-52-7, 589-98-0; both from Fluka, Steinheim, Germany) were applied to 1 cm-deep Teflon containers of 5 and 14 mm diameter, respectively. Flies were presented with both odours during training, but only one was paired with light for optogenetic activation (465 nm) or silencing (520 nm), whereas the other odour was presented alone (see electronic supplementary material, figure S2, for more details). The flies were then tested in a T-maze for their choice between the two odours. From the number of flies choosing each odour (#), the relative preference was calculated as

2.1 $$\mathrm{BA}\;\mathrm{Preference}\;=\left(\frac{\#{\;}_{\mathrm{BA}}-\#{\;}_{\mathrm{OCT}}}{\#\;\mathrm{Total}}\right)\times 100.$$

The presentation of BA and OCT with or without the light (*) was alternated between repetitions of the experiment, allowing an associative memory score to be obtained from reciprocally trained sets of flies as

2.2 $$\mathrm{Memory}\;\mathrm{score}=\frac{\mathrm{BA}\;\mathrm{Preferenc}e_{\mathrm{BA}*}-\mathrm{BA}\;\mathrm{Preferenc}e_{\mathrm{OCT}*}}{2}.$$

Data were analysed with Kruskal–Wallis tests (KW-tests) to compare more than two groups, Mann–Whitney *U*-tests (*U*-test) for pairwise comparisons, one-sample sign-tests for comparisons to chance level (i.e. zero), in all cases with Bonferroni–Holm corrections of *p* < 0.05 significance levels as appropriate, using Statistica 11.0 (StatSoft, Hamburg, Germany) and R 2.15.1 (www.r-project.org).

## 3. Results

Presenting an odour together with optogenetically silencing MBON-11 via the green-light-gated anion-channel GtACR1 established aversive memory for the odour (figure 1*b*). This effect was replicated using three training cycles (figure 1*c*). Consideration of the genetic controls suggests a weak appetitive olfactory memory through the pairing of odour with the green light, which is visible to the flies. Critically, relative to either genetic control, silencing MBON-11 had a punishing effect. Conversely, does activating MBON-11 have a rewarding effect?

**Figure 2.**
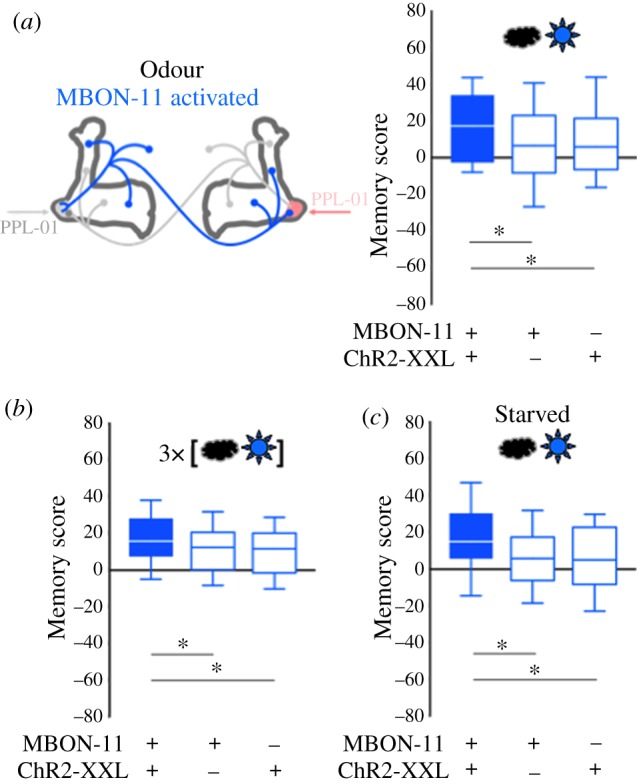
(*a*,*b*) The same as in figure 1*b*, *c* but using ChR2-XXL to activate MBON-11 (star). This leads to stronger appetitive learning in the experimental genotype than in genetic controls. (*c*) Same as the experiment in (*a*), but with an initial 18 h period of wet starvation, which improves appetitive learning [24]. Underlying preference scores can be found in electronic supplementary material, figure S3. Sample sizes and statistical results can be found in electronic supplementary material, table S1. Other details as in the legend of figure 1. (Online version in colour.)

Presenting an odour together with optogenetically activating MBON-11 via the blue-light-gated cation-channel ChR2-XXL established appetitive memory for the odour (figure 2). Corresponding to what is typically observed for primary food rewards such as sugar [24], this appetitive memory appeared slightly stronger under starved conditions (figure 2*c*; indeed starvation was shown to facilitate MBON-11 activity [25]). In the case of blue light too, the data from the genetic controls suggest a weakly rewarding effect. We further note that relative to the respective genetic controls, the punishing effect of silencing MBON-11 (figure 1*c*) appears to be stronger than the rewarding effect of activating it (figure 2*b*).

We conclude that silencing/activating MBON-11 has a punishing/rewarding effect.

## 4. Discussion

MBON-11 is GABAergic [13]. It targets premotor circuitry outside the mushroom bodies, and hetero-compartmental regions in the ipsi- and the contralateral mushroom body, and furthermore features a homo-compartmental and contralateral feedback loop onto the dopaminergic, punishing PPL1-01 neuron (figure 1*d*) [13,17,25,26]. All of these regions could contribute to reinforcement through manipulation of MBON-11 activity, and we expressly do not draw a conclusion as to which of these regions is indeed involved in these reinforcing effects. One scenario is that silencing MBON-11 lifts inhibition from PPL1-01, promotes PPL1-01 activity and thus exerts a punishing effect (but see [20]). Accordingly, the observation that activating MBON-11 has just a mild rewarding effect (figure 2) would suggest that spontaneous activity in PPL1-01 is moderate, and thus that silencing PPL1-01 would have less effect than activating it. Indeed, as previously reported, activating PPL1-01 is very strongly punishing (electronic supplementary material, figure S4B) [14,19], whereas silencing it is of no measureable rewarding effect (electronic supplementary material, figure S4C) (see [27] for a punishing effect of silencing the DAN of the γ3 compartment). This scenario would therefore suggest that targets other than PPL1-01 are responsible for the rewarding effect of activating MBON-11 (also see [20]).

Interestingly, the pathway from MBON-11 onto the glutamatergic MBON-01 neuron of the γ5 compartment and further from MBON-01 to the rewarding DANs of that compartment is critical for extinction learning after aversive training ([26]; also see [25]) (synonyms for MBON-01 are MBON-γ5β′2a and MB-M6). According to the scenario put forward in [26, fig. 7E-F], odours presented with MBON-11 silencing should lift inhibition from MBON-11 to MBON-01 and should thus drive the rewarding DANs of the γ5 compartment. This indirect, hetero-compartmental connection would thus support appetitive learning through MBON-11 silencing, whereas aversive learning would result for odours presented with MBON-11 activation—which is the *opposite* of what we report here! To reconcile this contradiction, consider that during second-order conditioning a stimulus X is first paired with primary reinforcement, and then X is presented together with a novel stimulus A in the absence of primary reinforcement. Whereas during AX training the effects of X as a reinforcement-predicting, second-order reinforcer will initially dominate, extended AX training will extinguish the X-with-reinforcement association. The above scenario would thus suggest that the opposing effects of second-order reinforcement and extinction learning, well known to practitioners of this paradigm, are related to homo- versus hetero-compartmental processes.

We note that placing the behavioural effects of manipulating MBON-11 activity into an experimental psychology framework of secondary reinforcement processing also encompasses the effect labelled ‘BGAM’ (for blockade of MBON-γ1pedc-induced aversive memory) [20, fig. 3B,C], obtained by blocking synaptic output from MBON-11 (also see [28]). Critically, the present framework suggests that silencing MBON-11 or preventing synaptic output from it leads to aversive learning about the odour paired with such treatment, whereas [20, p. 569] suggests that synaptic output from MBON-11 is necessary to prevent aversive learning about odours presented in an unpaired manner (for a discussion of paired and unpaired learning, see [29]).

We think that it is interesting that activity in a cell such as MBON-11 can be an analogue of second-order reinforcement, because this is the earliest site efferent to the memory trace in the presynaptic terminals of the mushroom body KCs for such an effect. This might inform the search for such analogues of secondary reinforcement in other species. It also raises the question of how much further down efferent pathways such analogues of second-order reinforcement can be observed, and indeed what the relation of action to valence is.

## Supplementary Material

Figure S1

## Supplementary Material

Figure S2

## Supplementary Material

Figure S3

## Supplementary Material

Figure S4

## Supplementary Material

Table S1

## Data accessibility

For data and statistical report, see the electronic supplementary material, table S1.
