# Supplementary figures and images for: An optogenetic analogue of second-order reinforcement in Drosophila
Source: Biol Lett. 2019 Jul 3;15(7):20190084. doi: 10.1098/rsbl.2019.0084 (PMC6684970; doi:10.1098/rsbl.2019.0084)

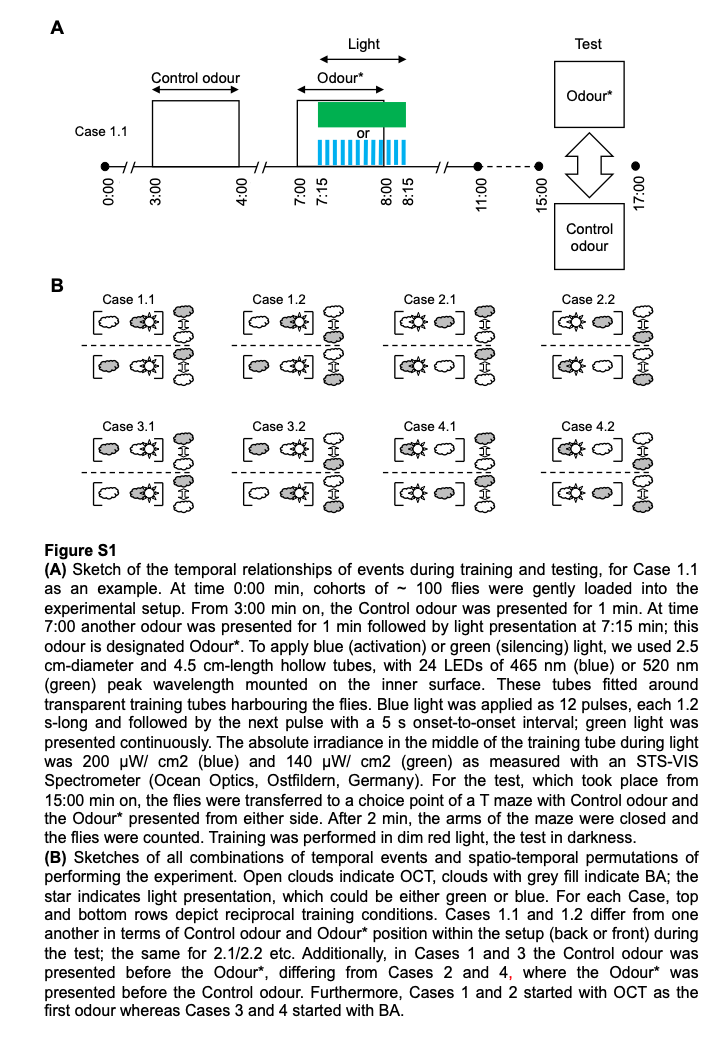

Supplement: Figure S1 [file rsbl20190084supp1.tiff]

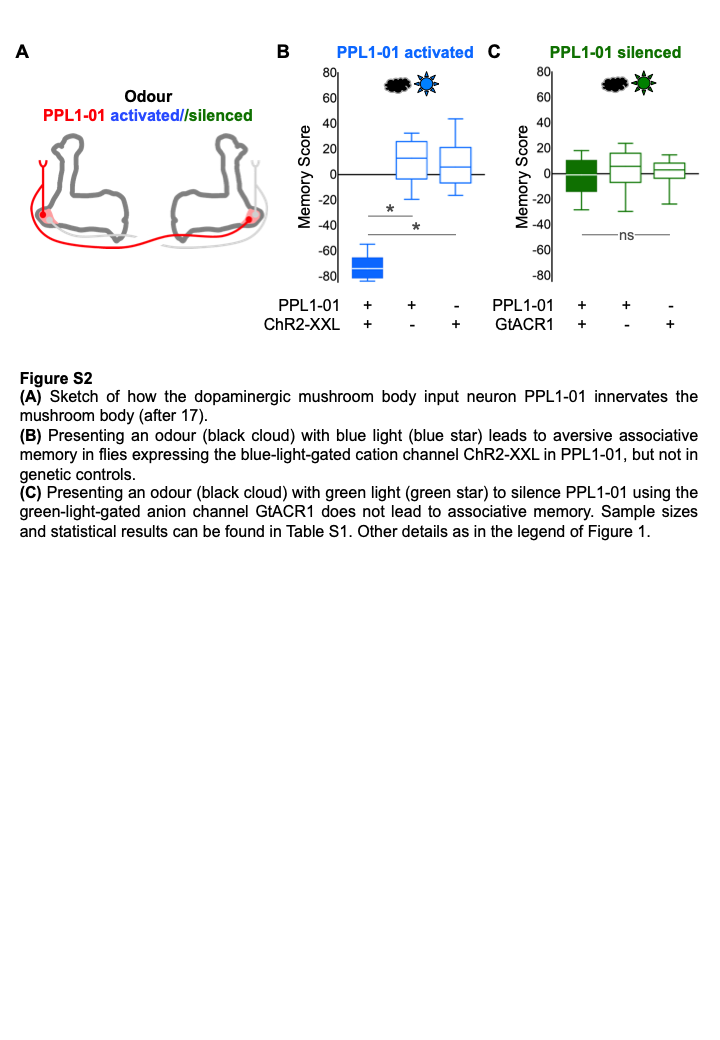

Supplement: Figure S2 [file rsbl20190084supp2.tiff]

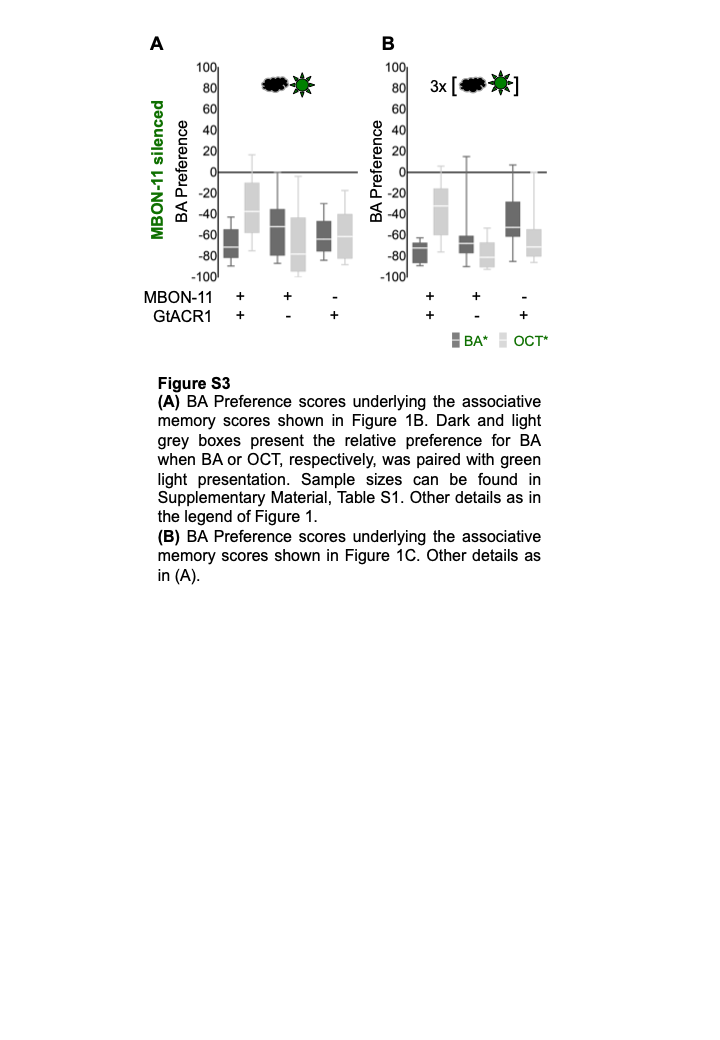

Supplement: Figure S3 [file rsbl20190084supp3.tiff]

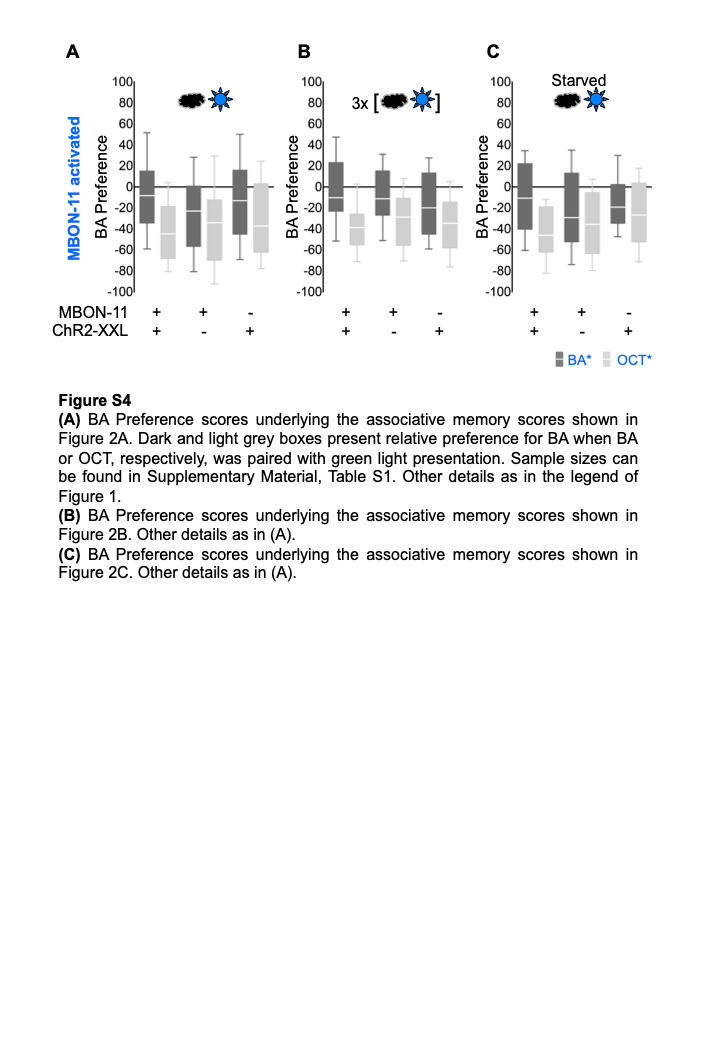

Supplement: Figure S4 [file rsbl20190084supp4.tiff]
